# Microglia derived from human induced pluripotent stem cells are regulated by osteopontin, an endogenous extracellular matrix protein maintaining immune homeostasis

**DOI:** 10.3389/fnins.2026.1785992

**Published:** 2026-06-24

**Authors:** Daniel Navin Olschewski, Anna Maria Koenig, Helena Mira Nerone, Elena Gross, Tomo Šarić, Sabine Ulrike Vay, Michael Schroeter, Gereon R. Fink, Maria Adele Rueger

**Affiliations:** 1 Cognitive Neuroscience, Institute of Neuroscience and Medicine (INM-3), Research Center Juelich, Juelich, Germany; 2 Department of Neurology, University of Cologne, Faculty of Medicine and University Hospital Cologne, Cologne, Germany; 3 Center for Physiology and Pathophysiology, Institute for Neurophysiology, Faculty of Medicine and University Hospital Cologne, University of Cologne, Cologne, Germany

**Keywords:** *in vitro* model culture system, iPSC (induced pluripotent stem cell), microglia, neuroinflammation, osteopontin

## Abstract

**Introduction:**

Microglia are brain-resident immune cells responsible for maintaining homeostasis, coordinating responses to injury and disease, and mediating regeneration. Upon activation, they undergo dynamic changes in morphology, gene expression, and function, reflecting the nature and context of the stimuli encountered. Although pharmacological modulation of microglia holds great promise for treating various neurological disorders, its development is hampered by a major translational roadblock: Human microglial cell lines commonly used in preclinical studies, as well as primary rodent microglia, substantially limit the translatability of results. Here, we aimed to generate microglia from human induced pluripotent stem cells (hiPSCs) and to demonstrate their physiological responsiveness to the brain-endogenous, context-relevant ligand osteopontin (OPN).

**Materials and methods:**

Microglia generated from two healthy hiPSC lines were stimulated with OPN, lipopolysaccharide (LPS), or their combination for 24 h and subsequently analyzed. Microglial identity and the expression of the phagocytic cell marker cluster of differentiation 68 (CD68) were determined by immunocytochemistry. Cell viability was assessed by propidium iodide (PI)/Hoechst staining, morphological activation was evaluated using Sholl analysis, and inflammatory gene expression changes were assessed by RT-qPCR.

**Results:**

hiPSC-derived microglia acquired a native central nervous system (CNS)-specific immunophenotype, expressing the microglia-specific markers ionized calcium-binding adapter molecule 1 (IBA1), transmembrane protein 119 (TMEM119), PU.1, and Spalt-like transcription factor 1 (SALL1), while remaining negative for Myb and membrane-spanning 4-domains, subfamily A, member 7 (MS4A7) at the protein level. Exposure to LPS led hiPSC-derived microglia to adopt a rounded, process-retracted shape and to increase CD68 protein intensity, a surrogate marker of lysosomal and phagocytic activity, while downregulating the anti-inflammatory marker cluster of differentiation 206 (CD206) at the transcriptional level. OPN induced a distinct microglial functional state characterized by intermediate morphology, increased CD68 intensity, and reduced homeostatic gene expression, without eliciting robust inflammatory gene expression. Intriguingly, OPN prevented LPS-induced microglial cell death, and when hiPSC-derived microglia exposed to LPS were additionally treated with OPN, the morphological effects of LPS were reversed.

**Conclusion:**

OPN induced a distinct early response profile in hiPSC-derived microglia, characterized by intermediate morphological remodeling, increased CD68 intensity, and reduced homeostatic gene expression, without overt pro-inflammatory gene expression. These findings support the role of OPN as a physiological priming signal in microglia and highlight hiPSC-derived microglia as a model for studying regulators of microglial modulation.

## Graphical abstract

**Figure:**
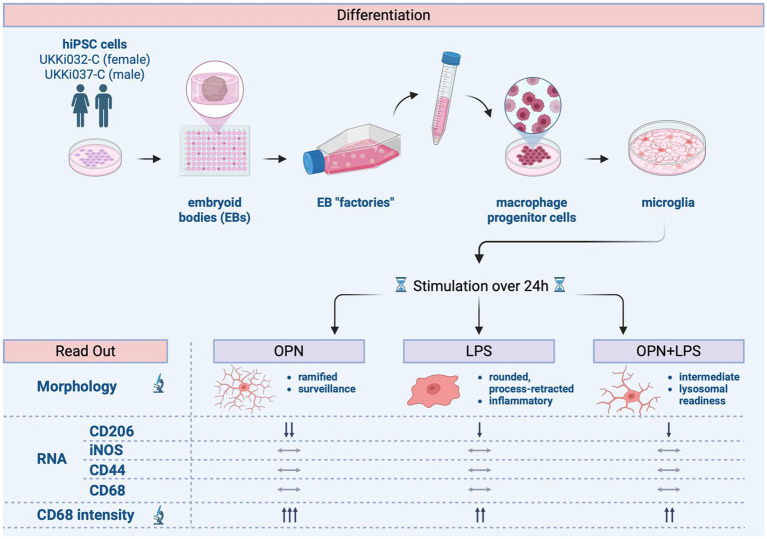
Human induced pluripotent stem cells (hiPSCs) generated from peripheral blood mononuclear cells (PBMCs) of one female and one male healthy donor were differentiated into microglia via embryoid body (EB) and macrophage progenitor stages. After 10 days of maturation, hiPSC-derived microglia were exposed to lipopolysaccharide (LPS), osteopontin (OPN), or their combination for 24 h. Cell survival, phenotypic maturation, microglial morphology, and immunological responses were assessed using a cell viability assay, immunocytochemistry including cluster of differentiation 68 (CD68) intensity levels and ramification quantification (via Sholl analysis), and RT-qPCR for selected inflammatory genes. Graphical abstract created with BioRender.

## Introduction

Microglia are the resident immunocompetent macrophages of the central nervous system (CNS), responsible for maintaining homeostasis, surveying the brain parenchyma, and coordinating responses to injury and disease. Upon activation, microglia undergo dynamic changes in morphology, gene expression, and function that reflect both the nature and context of the stimuli encountered (Davalos et al., 2005; Nimmerjahn et al., 2005; Kettenmann et al., 2011). In recent years, the diversity of microglial states has been highlighted beyond the binary M1/M2 polarization paradigm, revealing a spectrum of heterogeneous phenotypes with distinct transcriptional, metabolic, and disease-related signatures (Ransohoff, 2016; Vay et al., 2018; Stratoulias et al., 2019; Etebar et al., 2026; Paolicelli et al., 2022; Martins-Ferreira et al., 2025; Tuddenham et al., 2024).

Although rodent models have greatly advanced our understanding of microglia, species-specific differences in gene expression, signaling pathways, and developmental origins still limit the translatability of these findings to humans (Galatro et al., 2017; Gosselin et al., 2017). Immortalized human microglial cell lines have been shown to differ in marker expression, morphology, and function, most likely because the artificial transformation process disrupts physiological cell regulation (Woolf et al., 2025). In recent years, human induced pluripotent stem cell (hiPSC)-derived microglia have emerged as a promising model system. They recapitulate key features of brain-resident microglia, including functional properties such as phagocytosis and cytokine release (Muffat et al., 2016; Abud et al., 2017). However, generating human microglia from hiPSCs is complex, and their full capacity remains incompletely understood. Specifically, it is unknown whether and how hiPSC-derived microglia respond to brain-endogenous physiological cues.

Osteopontin (OPN), also known as secreted phosphoprotein 1 (SPP1), is an endogenous phosphoglycoprotein expressed by multiple CNS-resident cells, including neurons and glial cells (Kunii et al., 2009; Vay et al., 2021). OPN signals in microglia through cluster of differentiation 44 (CD44) and integrin receptors and plays a pivotal role in various CNS diseases in restoring immune homeostasis, for example, by modulating inflammatory responses, promoting repair, supporting neuroprotection, and restoring the blood–brain barrier. Highly increased levels of OPN have been observed in the acute phase after ischemic stroke and brain injury (Schroeter et al., 2006; Choi et al., 2007; Wang and Denhardt, 2008; Brown et al., 2011; Gliem et al., 2015; Riew et al., 2019; Cappellano et al., 2020; Vay et al., 2021; Sun et al., 2024). After a stroke, infiltrating macrophages and resident microglia induce OPN expression as part of a coordinated inflammatory and reparative response (Gliem et al., 2015; Riew et al., 2019; Vay et al., 2021). Despite its relevance, the direct effects of OPN on human microglial morphology and gene expression, especially compared to prototypical inflammatory stimuli, such as LPS, have not yet been elucidated.

In this study, we used a robust differentiation protocol to generate hiPSC-derived microglia and evaluate their responses to OPN, both alone and in combination with LPS. Using a combination of viability assays, immunofluorescence, Sholl analysis, CD68 quantification, and RT-qPCR, we assessed whether and how OPN modulates microglial morphology, CD68 expression, and gene expression.

## Materials and methods

### Human iPSC culture and differentiation

In total, two healthy Caucasian hiPSC lines were used: UKKi032-C (NP0141-31B, female; P34–P49) and UKKi037-C (NP0144-41, male; P29–P36). They were generated from peripheral blood mononuclear cells by the Saric group and have been previously fully characterized (Hamad et al., 2019; Chirico et al., 2022). The cell lines are part of the European Bank for induced pluripotent Stem Cells (EBiSC, https://ebisc.org/) and are registered in the online registry for human PSC lines, hPSCreg (https://hpscreg.eu/). All experiments were conducted according to the criteria of the code of proper use of human tissue used in Germany. The two hiPSC lines were cultured as previously described by Hamad et al. (2019). Briefly, hiPSC lines were cultured in E8 medium composed of DMEM/F12 (1:1) + GlutaMAX (Thermo Fisher Scientific, cat# 31331-028) supplemented with 64 μg/mL L-ascorbic acid phosphate magnesium n-hydrate (Wako Chemicals Europe, cat# 013-12061), 20 μg/mL insulin (Lilly Deutschland GmbH, “Humalog 100 I.E.”), 5 μg/mL transferrin (Sigma-Aldrich, cat# T3705), 14 ng/mL sodium selenite (Sigma-Aldrich, # S5261), 100 ng/mL heparin sodium salt (Sigma-Aldrich, cat# H3149), 100 ng/mL fibroblast growth factor 2 (FGF-2, Peprotech, cat# 100-18B), and 2 ng/mL transforming growth factor *β* (TGF-β, Peprotech, # 100-21). Cells were grown on 6-well plates (BD, cat#734-0019), which were coated for 1 h at room temperature with the recombinant human protein vitronectin (VTN-N; Thermo Fisher Scientific, cat# A14700) diluted at 1% with DPBS (Invitrogen, cat# 14190094) and incubated in a humidified incubator at 37 °C with 5% CO_2_. Medium was changed every other day, and cells were passaged every 3–4 days at a 1:3 to 1:20 split ratio using Versene (Thermo Fisher Scientific, cat# 15040066). Following dissociation of hiPSCs, E8 medium was supplemented with 5 μM Rho Kinase (ROCK) inhibitor (Y-27632, Sigma Aldrich, cat# 688000) for the first day after passaging.

### Generation of hiPSC-derived microglia

The two aforementioned hiPSC lines were used to generate hiPSC-derived microglia (van Wilgenburg et al., 2013; Haenseler et al., 2017). Briefly, to generate uniform embryonic bodies (EBs), hiPSCs were dissociated at 80–90% confluency using Accutase (1 mL/10 cm^2^, Thermo Fisher Scientific, cat# A1110501) for 5 min at 37 °C, followed by centrifugation. The dissociated hiPSCs were resuspended in mTeSR-1 (Stem Cell Tech, cat# #85850), the ROCK inhibitor (Y-27632, 1 mM, Sigma Aldrich, cat# 688000), bone morphogenetic protein 4 (BMP-4; 50 ng/mL, Miltenyi Biotec, cat# 130-110-921), stem cell factor (SCF; 20 ng/mL, Miltenyi Biotec, cat# 130-093-991), and vascular endothelial growth factor (VEGF; 50 ng/mL, Peprotech, cat# 100-20). The addition of the three growth factors—BMP-4, VEGF, and SCF—was required during the initial EB formation stage to induce hematopoiesis (van Wilgenburg et al., 2013).

hiPSCs were seeded at 10,000 cells/well into a round-bottom, low-attachment 96-well plate (Corning, 7007). To ensure uniform hiPSC aggregation into EBs, brief centrifugation (3 min at 100 g) of the 96-well plate was performed (van Wilgenburg et al., 2013). A full medium change without the ROCK inhibitor was conducted on day 2. From day 4 onward, EBs underwent directed differentiation. Approximately 40 EBs were transferred into a T25 cell culture flask containing the following media: X-Vivo 15 (Lonza, cat# BE04-418), GlutaMAX (2 mM, Life Tech, cat#. 35050-061), penicillin/streptomycin (100 U/mL, Life Tech, cat# 17502-048), and B-mercaptoethanol (50 μM, Life Tech, cat# 31350-010). In addition, for directed differentiation along the myeloid lineage, the growth factors interleukin-3 (IL-3, 25 ng/mL, Miltenyi Biotec, cat# 130–093-908) and recombinant human macrophage colony-stimulating factor (M-CSF, 100 ng/mL, Miltenyi Biotec, cat# 130-093-963) were added (van Wilgenburg et al., 2013). To ensure adhesion of the EBs, each T25 cell culture flask was incubated prior to EB transfer with 5% Matrigel diluted in DMEM/F12 (1:1) + GlutaMAX (Thermo Fisher Scientific, cat# 31331–028) for 3 h at room temperature. After the transfer of the EBs, the cell culture flask was left undisturbed for 1 week, followed by ¾ media changes every 4–5 days. Macrophage precursors were visible in the supernatant after 2–3 weeks, with the first harvest conducted after 1 month of growth. After harvest, the cells were maintained in a “microglia differentiation medium,” which was replaced every 3 days and consisted of advanced DMEM/F12 (Life Tech, cat# 12634-010), N2 supplement (1x, Life Tech, 17502-048), GlutaMAX (2 mM), B-mercaptoethanol (50 μM), penicillin/streptomycin (50 U/mL), interleukin-34 (IL-34, 100 ng/mL, Miltenyi Biotec, cat# 130-105-780), and granulocyte-macrophage colony-stimulating factor (GM-CSF; 10 ng/mL, Miltenyi Biotec, cat# 130-093-862). Maturation into hiPSC-derived microglia was completed after 10 days in culture.

### Treatment of hiPSC-derived microglia

A total of 10 days after seeding, hiPSC-derived microglia were incubated with either OPN at 6.25 ng/mL, 12.5 ng/mL, and 25 ng/mL (Sigma Aldrich, cat# SRP3131); LPS at 1 ng/mL, 5 ng/mL, and 10 ng/mL; or a combination of OPN 12.5 ng/mL + LPS 5 ng/mL for 24 h. Consequently, RNA was isolated, a live/dead assay was performed, and microglia were fixed with 4% paraformaldehyde (PFA) for immunocytochemical staining.

### Cell viability assay

The cell viability assay was performed to identify a concentration that induces robust immunological stimulation without overt cytotoxicity. It was also used to assess the potential co-stimulatory effect of OPN on cell viability.

Cell survival was assessed by staining dead cells with propidium iodide (PI, Thermo Fisher Scientific, cat# P1304MP), while all cells, regardless of viability, were counterstained with Hoechst 33342 (Thermo Fisher Scientific, cat# H3570), as previously described (Vay et al., 2021; Olschewski et al., 2024). A total of three images per well were captured using an inverted fluorescence microscope (Leica Microsystems, DMi8 inverted microscope with LAS X software). Both Hoechst-stained and propidium iodide-stained cells were counted automatically using Fiji (NIH ImageJ2, v.2.9.0). The ratio of propidium iodide-positive cells to the total cell count was used to determine the proportion of cell death. Experiments were performed in at least three independent biological replicates, each using an independent batch of hiPSC-derived microglia. For each biological replicate, two technical replicate wells were analyzed per condition. The resulting mean values ± SEM were established among equally treated samples.

### Immunocytochemical staining

Markers of hiPSC pluripotency in culture were determined by immunocytochemistry. Cells were fixed with 4% paraformaldehyde (PFA) and stained for tumor rejection antigen 1–81 (TRA-1-81; mouse, dilution 1:100, cat# sc-21706, RRID:AB_628386, Santa Cruz Biotechnologies), OCT3/4 (goat, dilution 1:200, cat# sc-5279, RRID:AB_628051, Santa Cruz Biotechnologies), NANOG (rabbit, dilution 1:200, cat# 4903-S, RRID:AB_10559205, Cell Signaling Technology), and stage-specific embryonic antigen 4 (SSEA4; mouse, dilution 1:200, cat# Sc-21704, RRID:AB_628289, Santa Cruz Biotechnologies). OCT3/4 and NANOG are transcription factors essential for maintaining pluripotency and self-renewal, while TRA-1-81 and SSEA4 serve as surface expression markers of undifferentiated human pluripotent stem cells (Pan and Thomson, 2007; Oda et al., 2010; Zhao et al., 2012; Sivasubramaniyan et al., 2015).

To differentially evaluate the cells for microglial characteristics, we stained hiPSC-derived microglia for ionized calcium-binding adapter molecule 1 (IBA1; rabbit polyclonal antibody, dilution 1:500, cat# 019-19741, RRID:AB_839504, FUJIFILM WAKO / chicken monoclonal, dilution 1:500, cat# ab318302, abcam), transmembrane protein 119 (TMEM119; rabbit polyclonal antibody, dilution 1:200, cat# ab185333, RRID:AB_2687894, abcam), Spalt-like transcription factor 1 (SALL1; mouse monoclonal, dilution 1:50, Cat# PP-K9814-00, RRID:AB_2183228, R and D Systems), PU.1 (rabbit monoclonal, dilution 1:200, Cat# HM2258, RRID:AB_10679352, Hycult Biotech), membrane-spanning 4-domains, subfamily A, member 7 (MS4A7; rabbit polyclonal, dilution 1:50, Cat# HPA017418, RRID:AB_1854137, Sigma-Aldrich), and Myb (mouse monoclonal, dilution 1:200, (Millipore Cat# 05-175, RRID:AB_11213983; Ito et al., 1998; Banerjee et al., 2020; Cakir et al., 2022; Ruan and Elyaman, 2022; Fixsen et al., 2023). CD68 (mouse monoclonal, dilution 1:200, Cat# ab955, RRID:AB_307338, abcam) was used as a surrogate marker to assess lysosomal marker abundance and phagocytic capacity. For visualization, AlexaFluor-labeled anti-mouse IgG, anti-rabbit IgG, or anti-chicken IgY secondary antibodies were used (dilution 1:300, Invitrogen, Karlsruhe, Germany); all cells were additionally counterstained with Hoechst 33342 (Life Technologies, Darmstadt, Germany). Representative images were obtained using an inverted fluorescence microscope (Leica Microsystems, DMi8, with LAS X software) or a confocal microscope (LSM710, Carl Zeiss, Germany).

To quantify microglial marker expression, immunofluorescence images were analyzed using the Cell Counter plugin in Fiji/ImageJ. For each image, the total number of Hoechst-positive nuclei and the number of marker-positive cells (IBA1, TMEM119, PU.1, or SALL1) were counted manually. The percentage of marker-positive cells was calculated per image. As hiPSC-derived microglia occasionally contained binucleated or trinucleated cells, total cell counts based on Hoechst-positive nuclei may slightly overestimate actual cell numbers, resulting in conservative estimates of marker positivity. Quantification was performed across two independent differentiations per hiPSC line.

### THP-1 macrophages

To validate antibody specificity for Myb and MS4A7, THP-1 monocytes were differentiated into macrophage-like cells. Briefly, THP-1 cells were seeded and treated with 25 ng/mL phorbol 12-myristate 13-acetate (PMA) for 24 h in RPMI medium (cat# 31870-025) supplemented with 10% heat-inactivated fetal bovine serum, 1% penicillin/streptomycin, and 2 mM glutamine. After PMA treatment, cells were washed twice with medium and maintained for an additional 48 h to allow recovery and differentiation into macrophage-like cells. The cells were then fixed with 4% paraformaldehyde for 10 min and stained under identical conditions as hiPSC-derived microglia. THP-1 macrophages were additionally stained for PU.1 and SALL1 to provide a comparative marker profile between peripheral macrophages and hiPSC-derived microglia.

### Sholl analysis

**Figure 1:**
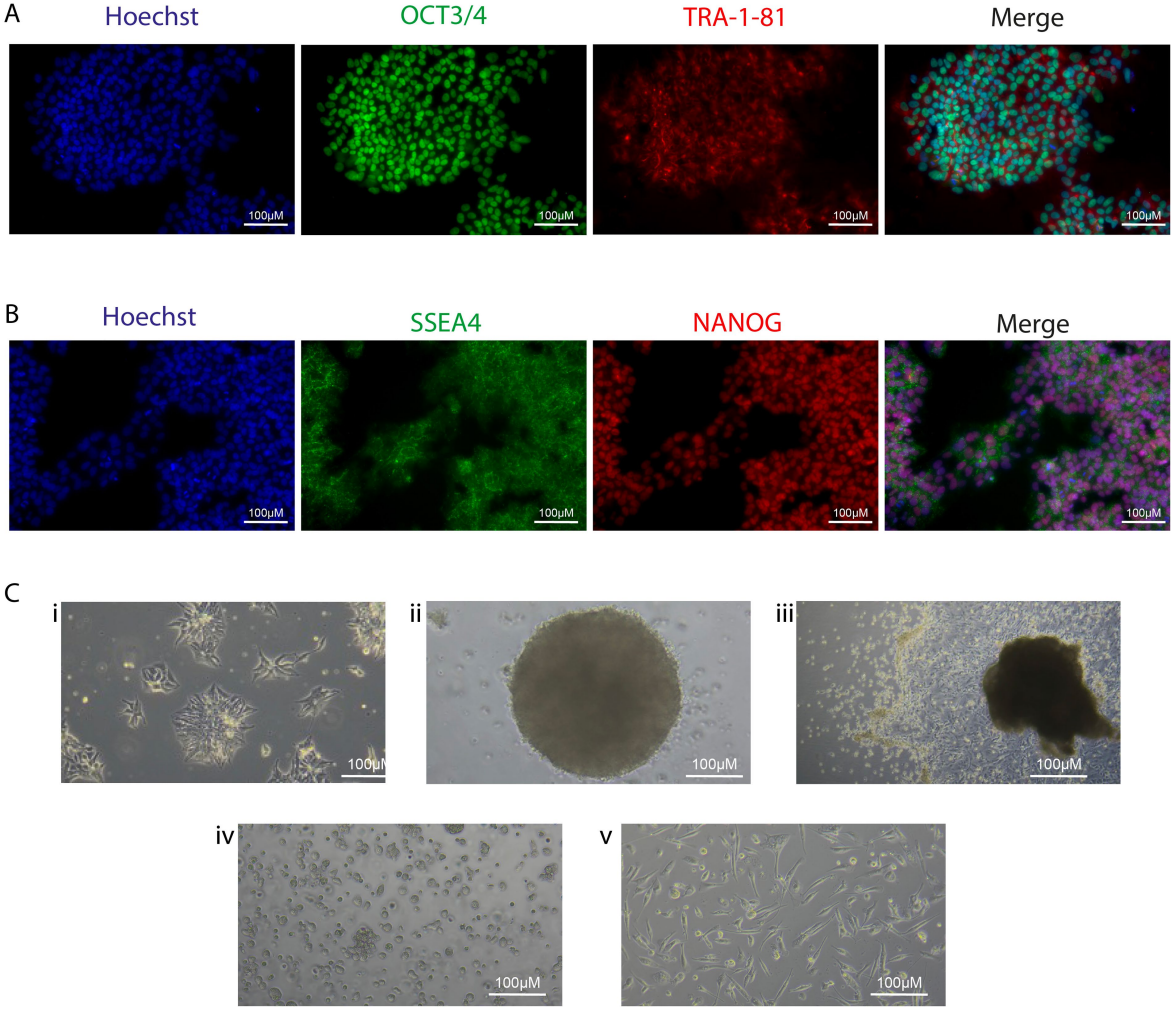
Human induced pluripotent stem cells (hiPSCs) expressed canonical markers of pluripotency **(A)** Representative immunofluorescence images of hiPSCs stained positive for OCT3/4 (green), a nuclear transcription factor essential for maintaining pluripotency, and TRA-1-81 (red), a surface antigen marking undifferentiated human pluripotent stem cells. **(B)** Similarly, hiPSCs stained positive for SSEA-4 (green), a glycoprotein expressed on the surface of undifferentiated pluripotent cells, and NANOG (red), a nuclear transcription factor involved in the regulation of self-renewal. Hoechst (blue) was used to stain nuclei. **(C)** Representative brightfield images showing key stages of microglial differentiation from hiPSCs: (i) undifferentiated hiPSCs, (ii) embryoid bodies (EBs), (iii) EB-derived factory aggregates that continuously release myeloid progenitors, (iv) macrophage precursor cells harvested from EB factories, and (v) terminally differentiated microglia-like cells. Images illustrate the morphological progression from pluripotent stem cell colonies to mature, ramified microglia.

To assess differences in microglial morphology between the conditions, Sholl analysis was performed in ImageJ (NIH ImageJ2, v.2.9.0) using the Sholl analysis plugin (Gensel et al., 2010). For each condition, 25–30 IBA1-positive cells per biological replicate were recorded at a magnification of 40x with the Leica Microsystems microscope and subsequently analyzed. Image analysis was performed in a blinded manner. The soma center was defined as the origin of concentric circles, which were automatically drawn at 5 μm intervals to measure the number of intersections between cell processes and each circle, reflecting the degree of ramification. One representative hiPSC-derived microglial cell analyzed by Sholl analysis is depicted in Figure 1A.

Sholl profiles were generated for each cell, and the area under the curve (AUC) was calculated to provide a single numerical measure of overall branching complexity. For each individual cell, the area under the curve (AUC) of the Sholl profile was calculated using the trapezoidal rule, integrating the total number of intersections across the full radial distance. This yielded a single numerical value per cell representing overall branching complexity. AUC values were then grouped by treatment condition and analyzed statistically. The experiment was performed in at least three independent biological replicates, with three technical replicates per group. The resulting mean values ± SEM were calculated for each experimental group.

### CD68 intensity quantification

Quantification of CD68 signal intensity was conducted using RGB-overlay immunofluorescence images in Fiji/ImageJ (v1.53). Individual hiPSC-derived microglia were identified based on TMEM119-positive morphology, and regions of interest (ROIs) were manually defined around each cell. Mean CD68 fluorescence intensity was measured within each ROI and background-corrected by subtracting the mean intensity of a cell-free region in the same image. All images were recorded from four independent differentiations (two per hiPSC line), and intensity settings were kept unchanged across all conditions (Control, OPN, LPS, and OPN + LPS) and recordings. For each condition, approximately 18–20 cells per biological replicate were analyzed. To avoid pseudoreplication, CD68 intensity values were averaged per biological replicate (*n* = 4) for statistical analysis. All intensity values are reported in arbitrary units (a.u.). The resulting mean values ± SEM were calculated for each experimental group.

### RT-qPCR

RNA from hiPSC-derived microglia was isolated using the Qiagen RNeasy Plus Micro Kit (Qiagen, cat# 74034) according to the manufacturer’s instructions. RNA concentration and purity were evaluated using a NanoDrop spectrophotometer (Thermo Fisher Scientific). According to the manufacturer’s instructions, total RNA (10 ng) was converted to cDNA by reverse transcription using the QuantiTect Transcription Kit (Qiagen, Hilden, Germany). Primers were obtained from Biolegio (Nijmegen, The Netherlands); their respective sequences and PCR parameters are listed in Table 1. The samples were amplified and quantified using a LightCycler 96 system (Roche, Mannheim, Germany). PCR product integrity was evaluated using melting point analysis and agarose gel electrophoresis. The cycle threshold (CT) was normalized to housekeeping ribosomal protein L13a (RPL13a; ΔCT). Data are depicted as 2^(−ΔΔCT)^. RT-qPCR was performed in technical triplicates, and each experiment was conducted in at least three biological replicates. Mean values ± SEM were calculated for all samples in each experimental group.

**Table 1:** Primers and parameters of RT-qPCR.

RNA	Sequences forward/reverse 5′–3′	Temperature (°C) step 1/2/3	Duration (s) step 1/2/3	Accession number
iNOS	CTCCAATGTGACCTGGGACC/CTTGGCCATCCTCACAGGAG	95/63/72	15/30/30	NM_000625.4
CD206	CACCATCGAGGAATTGGACT/ACAATTCGTCATTTGGCTCA	95/56/72	15/30/30	NM_002438.4
CD68	GAGCCGAGAATGTCCACTGT/CACTGGGGCAGGAGAAACT	95/56/72	15/30/30	NM_001251.3
CD44	GGGATATCGCCAAACACCCA/TGGATGGCTGGTATGAGCTG	95/63/72	15/30/30	NM_000610.4
RPL13a	TGGTCGTACGCTGTGAAGG/AGGAAAGCCAGGTACTTCAACTT			NM_012423.4, isoform 1

### Statistical analysis

Microsoft Excel (Version 365, Microsoft Corp., USA) was used for processing raw numerical data. Representative images were edited in Fiji (NIH ImageJ2, v.2.9.0) by adjusting brightness, contrast, and sharpness for each color channel, applying minimal, non-significant modifications. Importantly, identical adjustment settings were applied uniformly across all images within each comparison group to ensure consistency and avoid introducing bias. For quantitative image analyses (CD68 intensity, marker quantification, and Sholl analysis), no brightness or contrast modifications were applied; raw fluorescence intensity values from unmodified images were used throughout. Figures were created using Adobe Illustrator (Adobe Inc., USA).

At least three technical replicates were carried out for the experiments with hiPSC-derived microglia. Statistical analyses and graphical visualization were conducted using GraphPad Prism (Version 10.5.0, GraphPad Software Inc., San Diego, CA, USA). For comparisons involving more than two groups, one-way analysis of variance (ANOVA) was used, followed by Tukey’s *post hoc* test. For Sholl analysis, two-way repeated measures ANOVA was conducted to evaluate the interaction between radial distance and treatment condition, followed by Dunnett’s T3 post hoc test to compare treatment groups with the control group. For Sholl AUC analysis, the Brown–Forsythe test and Welch’s ANOVA were used due to unequal variances, followed by Dunnett’s T3 post hoc test. Where applicable, variance assumptions were assessed using the Brown–Forsythe test and Bartlett’s test. Tukey’s post hoc test inherently controls the family-wise error rate for pairwise comparisons within each ANOVA. For comparisons across multiple independent endpoints, *p*-values were additionally evaluated using the Benjamini–Hochberg procedure to control the false discovery rate at 5%. Post-hoc power analyses were conducted using G*Power 3.1 for key ANOVA comparisons. Statistical significance is denoted as *p < 0.05, **p < 0.01, or ***p < 0.001.

## Results

### hiPSC expressed canonical pluripotency markers

To verify the pluripotent status of both hiPSC lines before differentiation, immunofluorescence staining for key transcriptional and surface markers was performed. OCT3/4 and NANOG, two transcription factors essential for maintaining pluripotency and self-renewal, were detected in both hiPSC lines (Figure 1A). In addition, robust surface expression of markers of undifferentiated human pluripotent stem cells, TRA-1-81 and SSEA4, was observed (Figure 1B). Representative pictures of all differentiation stages can be found in Figure 1C.

Our findings confirm a stable pluripotent phenotype of the hiPSC before directed differentiation, demonstrating its suitability for further experiments, including maturation into microglia.

### hiPSC-derived microglia acquired a native CNS-specific immunophenotype

**Figure 2:**
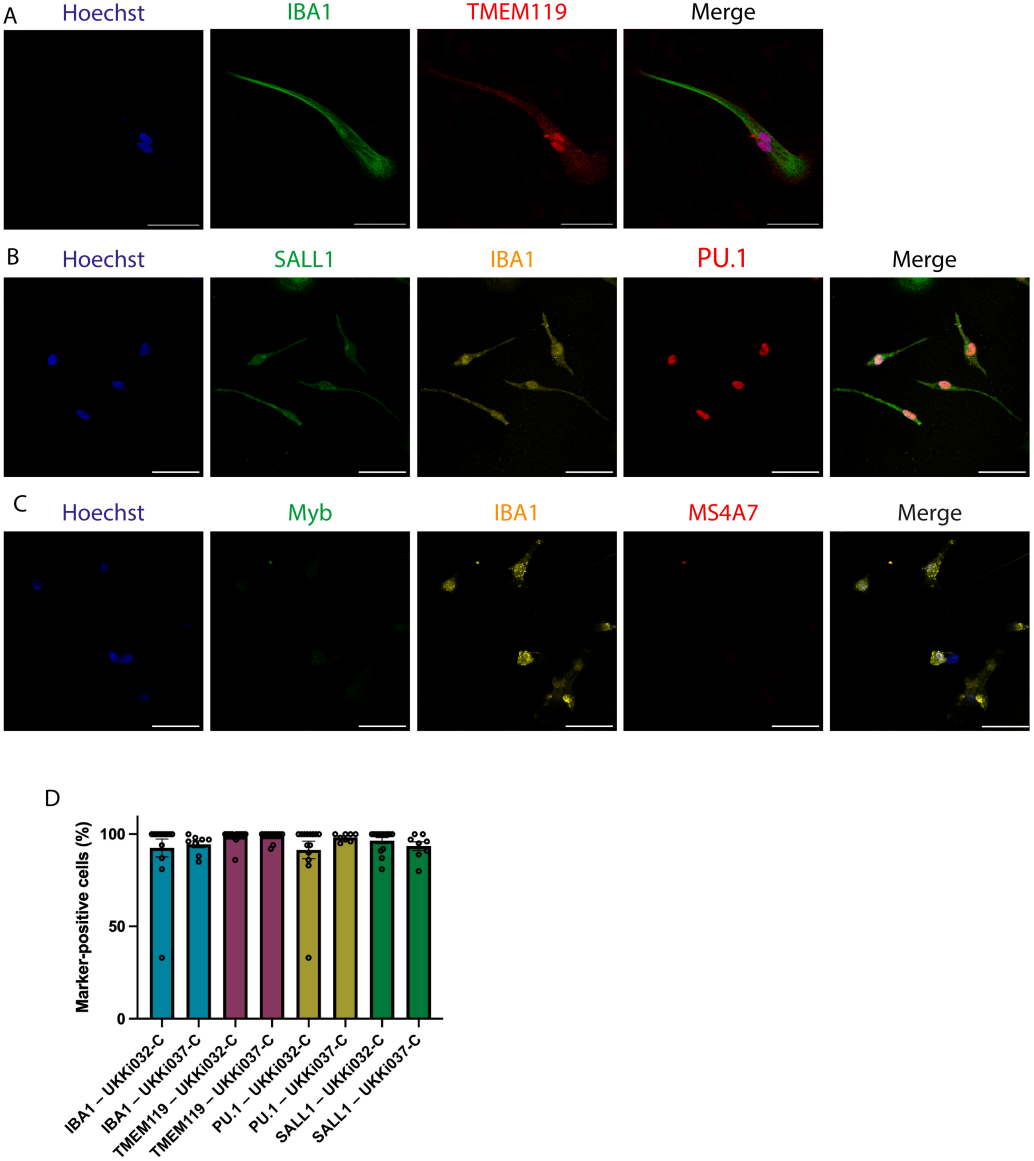
hiPSC-derived microglia acquired a native CNS-specific immunophenotype **(A)** Representative images of differentiated hiPSC-derived microglia co-expressing the typical microglial markers IBA1 (green) and TMEM119 (red). **(B)** IBA1 (yellow) co-localized with PU.1 (red) and SALL1 (green), distinguishing microglia from peripheral macrophages and neuroepithelial glia, respectively. **(C)** hiPCS-derived microglia did not express markers of peripheral macrophages such as Myb (green) and MS4A7 (red), validating their CNS-specific differentiation. Hoechst staining was used for nuclear visualization. Scale bars = 50 μm. **(D)** Quantification of microglial marker expression in hiPSC-derived microglia from both cell lines (UKKi032-C and UKKi037-C) across two independent differentiations per line. The percentage of marker-positive cells relative to total Hoechst-positive nuclei is shown for IBA1, TMEM119, PU.1, and SALL1. For UKKi032-C, 14 images (226 cells) were analyzed for PU.1, SALL1, and IBA1, and 15 images (223 cells) were analyzed for TMEM119. For UKKi037-C, eight images (262 cells) were analyzed for PU.1, SALL1, and IBA1, and 12 images (405 cells) were analyzed for TMEM119. Data are shown as mean ± SEM, with individual data points representing single images.

Following directed differentiation via EB and macrophage progenitor stages, hiPSC-derived microglia were characterized by immunofluorescence to assess lineage-specific marker expression, thereby validating microglial identity and ontogeny. The cells displayed robust positivity for the microglial markers IBA1, a cytoplasmatic myeloid marker widely used in the literature to identify microglia, and TMEM119, a transmembrane protein that stabilizes the microglial phenotype, maintaining homeostatic functions and potentially contributing to cell–cell communication and signal transduction (Figure 2A). Moreover, the microglia expressed PU.1, a transcription factor indicative of differentiation along the physiological myeloid lineage and mesodermal origin, in contrast to other glial cells originating from neuroepithelial tissue (Figure 2B). SALL1, a transcription factor associated with microglial maintenance that distinguishes microglia from peripheral macrophages, showed widespread intracellular distribution (Figure 2B). Importantly, hiPSC-derived microglia were negative for markers of a peripheral myeloid phenotype, such as the transcription factor Myb and the monocyte/macrophage marker MS4A7 (Figure 2C). To quantify microglial marker expression, immunofluorescence images from two independent differentiations per hiPSC line were analyzed. For UKKi032-C, a total of 226 cells across 14 images were assessed for PU.1, SALL1, and IBA1, and 223 cells across 15 images were assessed for TMEM119. For UKKi037-C, 262 cells across eight images were assessed for PU.1, SALL1, and IBA1, and 405 cells across 12 images were assessed for TMEM119. Across both lines and differentiations, hiPSC-derived microglia showed high and consistent expression of all four markers. TMEM119 positivity was 98.7 ± 1.0% (UKKi032-C) and 98.8 ± 0.8% (UKKi037-C). SALL1 was expressed in 96.5 ± 1.7% (UKKi032-C) and 93.6 ± 2.3% (UKKi037-C) of cells. IBA1 positivity was 92.5 ± 4.8% (UKKi032-C) and 94.5 ± 1.8% (UKKi037-C), and PU.1 was detected in 91.5 ± 4.7% (UKKi032-C) and 98.2 ± 0.7% (UKKi037-C) of cells. Marker expression was comparable between the two hiPSC lines and reproducible across independent differentiations, confirming robust and consistent microglial identity (Figure 2D).

To validate antibody specificity and confirm the absence of peripheral macrophage markers, THP-1 macrophages were stained under identical conditions. THP-1 cells were positive for PU.1, Myb, and MS4A7 and negative for SALL1, confirming the discriminatory power of the selected marker panel (Supplementary Figure 1).

Together, this marker profile demonstrated the CNS-specific, ontogeny-based differentiation of hiPSC-derived microglia and supported their distinction from peripheral macrophages and neuroepithelial glia.

### OPN maintained the viability of hiPSC-derived microglia under LPS exposure

**Figure 3:**
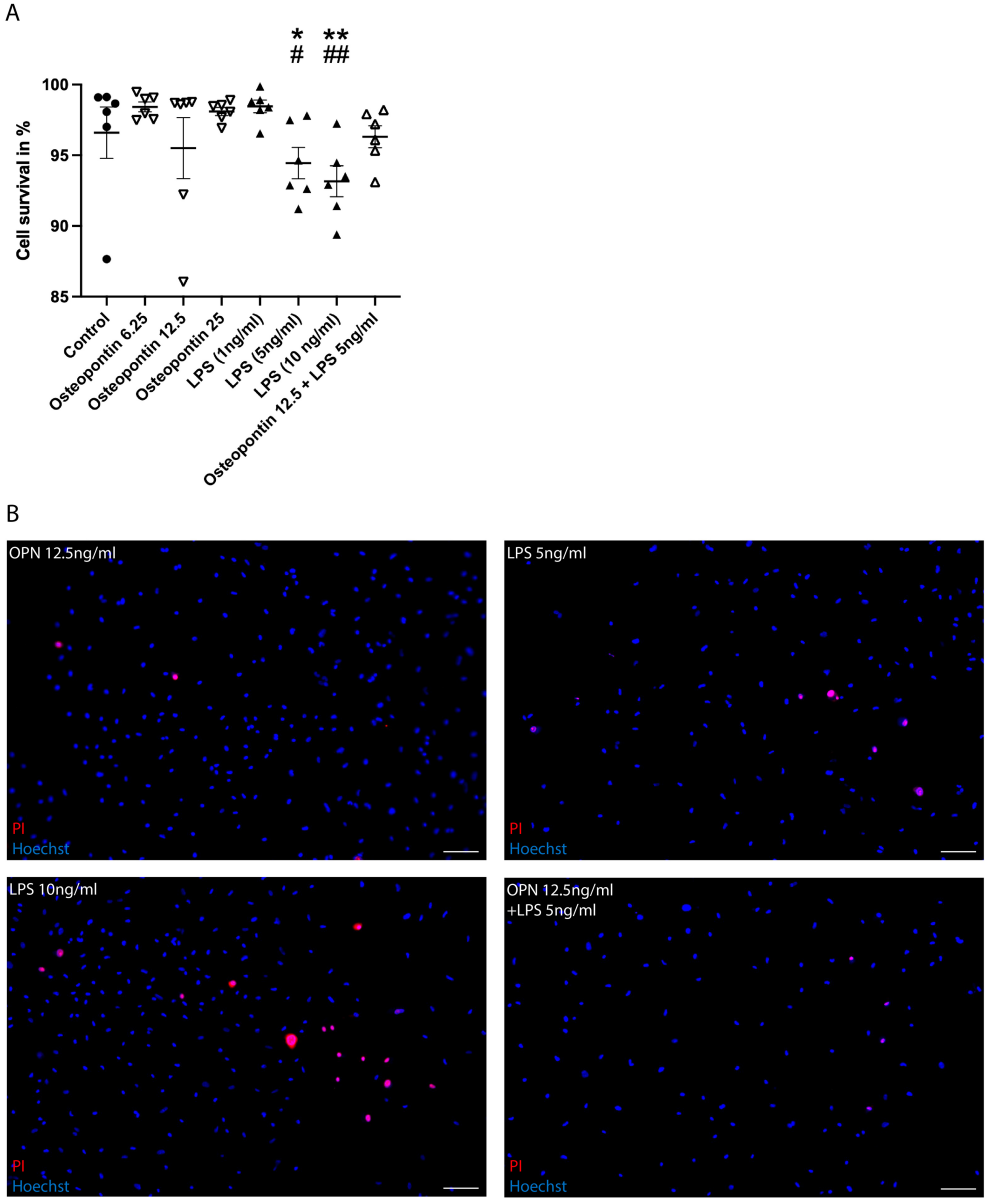
Effects of OPN and LPS on hiPSC-derived microglial viability **(A)** Quantification of cell viability using propidium iodide (PI) and Hoechst staining following 24 h treatment with OPN (6.25 ng/mL, 12.5 ng/mL, and 25 ng/mL), LPS (1 ng/mL, 5 ng/mL, and 10 ng/mL), or OPN (12.5 ng/mL) in combination with LPS (5 ng/mL). Cell viability was calculated as 100% minus the percentage of PI-positive nuclei relative to total Hoechst-positive nuclei. Data are shown as mean ± SEM from n = 3 biological replicates. Statistical analyses are described in the Results section. **(B)** Representative immunofluorescence images of PI (red) and Hoechst (blue) staining under selected treatment conditions. Scale bars = 100 μm.

To evaluate the effects of OPN and LPS on microglial viability, we conducted a live/dead assay using propidium iodide (PI) and Hoechst staining, after exposing the cells to the respective substances or their combination for 24 h (Figure 3). OPN alone did not affect microglial viability in any concentration tested (control vs. OPN 6.25–25 ng/mL, all *p* > 0.8; Figure 3A). On the other hand, the bacterial endotoxin LPS induced a slight, dose-dependent increase in cell death. While 1 ng/mL LPS had no effect on viability compared to the control (control vs. LPS 1 ng/mL: Δ ≈ −2%, *p* = 0.8080), treatment with 5 ng/mL LPS reduced viability from approximately 95% in control cultures to ~90% (control vs. LPS 5 ng/mL: Δ ≈ 5%, *p* = 0.6608). Exposure to 10 ng/mL LPS resulted in a slightly stronger reduction in viability to ~88–89% (control vs. LPS 10 ng/mL: Δ ≈ 7%, *p* = 0.1130), although this difference also did not reach statistical significance.

Co-treatment with OPN (12.5 ng/mL) did not significantly modify LPS-associated cytotoxicity, as microglial viability did not differ between cells treated with 5 ng/mL LPS alone and those treated with LPS plus OPN (LPS 5 ng/mL vs. LPS 5 ng/mL + OPN 12.5 ng/mL: Δ ≈ 2%, *p* = 0.8030). Importantly, viability under co-treatment conditions remained indistinguishable from untreated controls (control vs. OPN 12.5 + LPS 5 ng/mL: *p* > 0.9999). Based on these findings, we selected OPN at 12.5 ng/mL and LPS at 5 ng/mL for subsequent experiments to ensure cellular stimulation without compromising cell viability.

### hiPSC-derived microglia physiologically responded to inflammatory activation

To assess whether hiPSC-derived microglia were capable of a physiological response to activating stimuli, we analyzed morphological changes and CD68 expression as a surrogate marker of phagocytic activity.

**Figure 4:**
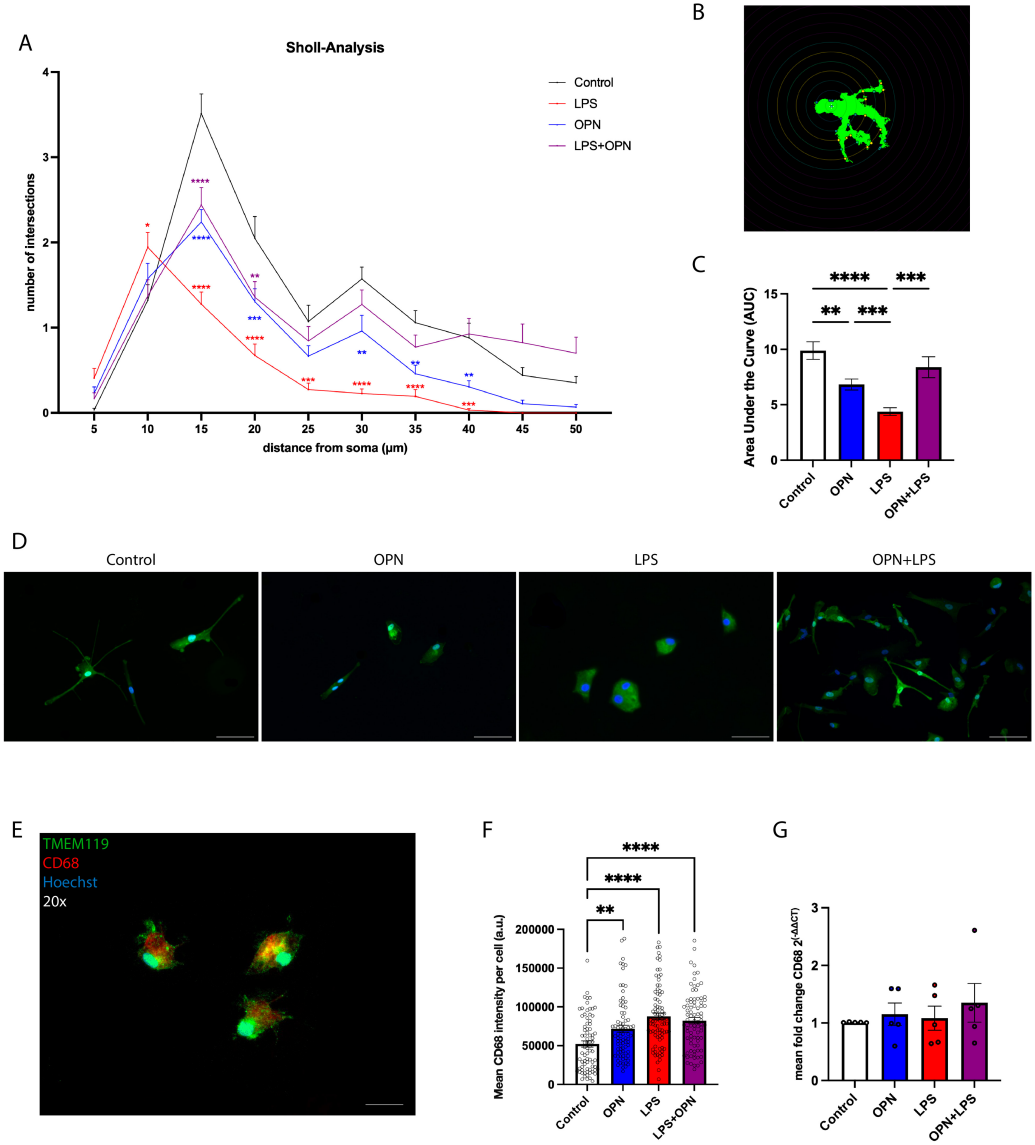
hiPSC-derived microglia physiologically responded to inflammatory activation Data are shown as mean ± SEM. Sample sizes and statistical analyses are described in the Results section. **(A)** Sholl analysis of hiPSC-derived microglial morphology following 24 h treatment with LPS (5 ng/mL), OPN (12.5 ng/mL), their combination, or untreated control conditions. The number of intersections was quantified as a function of distance from the soma. **(B)** Representative image of skeletonized microglial processes used for Sholl analysis. **(C)** Area under the curve (AUC) derived from the Sholl analysis shown in panel **(A)**, summarizing overall process complexity per condition. Due to unequal variances (Brown–Forsythe test, *p* < 0.001), the Brown–Forsythe test and Welch’s ANOVA were used, followed by Dunnett’s T3 *post hoc* test. *n* = 92–138 cells per condition from at least three independent biological replicates. **(D)** Representative immunofluorescence images of IBA1-stained (green) hiPSC-derived microglia following 24 h treatment with vehicle (Control), OPN (12.5 ng/mL), LPS (5 ng/mL), or their combination (OPN + LPS). Nuclei were counterstained with Hoechst (blue). Scale bar = 50 μm. **(E)** Representative immunocytochemical co-staining of hiPSC-derived microglia for TMEM119 (green) and CD68 (red); Hoechst (blue) nuclear counterstain. Scale bar = 50 μm. **(F)** Quantification of CD68 protein expression based on fluorescence intensity analysis. **(G)** Relative CD68 mRNA expression measured by RT-qPCR and normalized to RPL13a.

As shown in *in vivo* studies, microglia respond to certain stimulation by retracting their processes and adopting rounded, process-retracted morphology (Paolicelli et al., 2022). We quantified these morphological changes in our cultures via Sholl analysis. Specifically, the length of microglial processes from the soma was measured after rigid pre-processing steps (Figure 4B), resulting in a “number of intersections,” representing the number of processes exceeding a certain length, for each distance from the soma. Therefore, the shape of microglia under different treatment conditions could be visualized, quantified, and statistically compared across entire cultures (Figure 4A). Upon exposure to LPS (5 ng/mL), hiPSC-derived microglia retracted their processes and adopted rounded, process-retracted morphology, exactly mimicking the *in vivo* situation (Figure 4A, red line). In the presence of OPN (12.5 ng/mL), hiPSC-derived microglia also displayed reduced process complexity (Figure 4A, blue line) compared to cells under control conditions (Figure 4A, grey line). However, notably, the addition of OPN to hiPSC-derived microglia exposed to LPS (combinatory exposure) greatly reduced LPS-induced morphological effects on hiPSC-derived microglia (Figure 4A, purple line). We also present the Sholl data as the “area under the curve” (AUC; Figure 4C), highlighting the effects of the different treatments on microglial morphology. LPS clearly drove hiPSC-derived microglia toward rounded, process-retracted morphology (*p* < 0.0001 compared to the control). OPN alone also significantly reduced process complexity compared to the control (*p* = 0.0073). The combination of OPN and LPS did not differ significantly from the control (*p* = 0.7768) but differed significantly from LPS alone [*p* = 0.0006; Welch’s ANOVA, W(3, 234.0) = 18.18, *p* < 0.0001; post-hoc power: 99.5%; Figure 4C].

CD68 expression was assessed as a surrogate marker of lysosomal and phagocytic activity using a single-marker approach (Figure 4E). Both LPS (5 ng/mL) and OPN (12.5 ng/mL) alone, as well as their combination, significantly increased CD68 expression at the protein level, as measured immunocytochemically using fluorescence intensity analysis [one-way ANOVA, *F*(3, 320) = 13.46, *p* < 0.0001; post-hoc power: >99.9%; Figure 4F]. However, at the RNA level, no statistically significant changes in CD68 expression were observed, suggesting a mechanism independent of transcription (Figure 4G).

### CD206 was regulated in hiPSC-derived microglia

**Figure 5:**
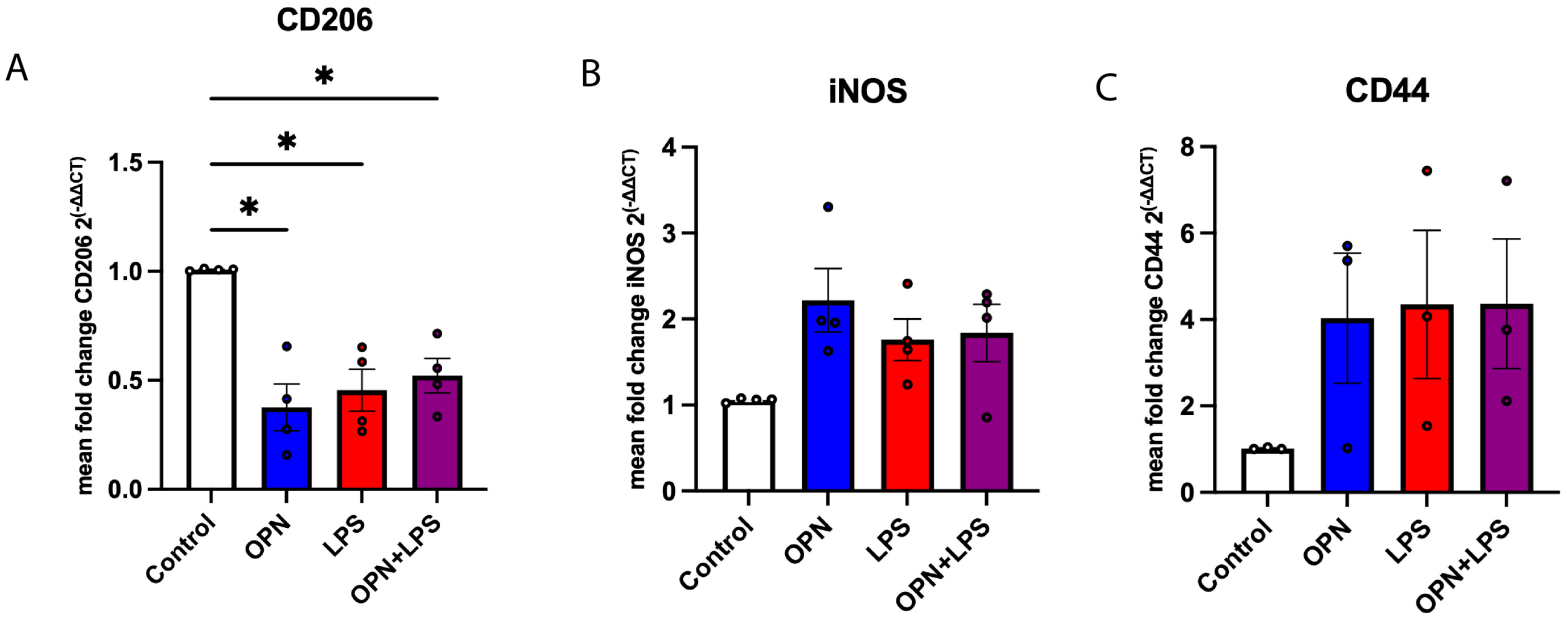
Relative mRNA expression of CD206, iNOS, and CD44 in hiPSC-derived microglia treated with vehicle (Control), OPN (12.5 ng/mL), LPS (5 ng/mL), or their combination mRNA levels of CD206 **(A)**, iNOS **(B)**, and CD44 **(C)** in hiPSC-derived microglia were determined by RT-qPCR and normalized to RPL13a. Data are shown as mean fold change [2^(−ΔΔCT)^] ± SEM from *n* = 4 biological replicates. For CD206, the Brown–Forsythe test and Welch’s ANOVA were used due to unequal variances, followed by Dunnett’s T3 post hoc test. For iNOS and CD44, one-way ANOVA with Tukey’s post hoc test was used. *p < 0.05, **p < 0.01, ***p < 0.001.

To determine the immunological status of hiPSC-derived microglia, we assessed gene expression of inducible nitric oxide synthase (iNOS), cluster of differentiation 206 (CD206), and CD44 following stimulation (Figure 5). CD206, a mannose receptor associated with mannose-mediated ligand uptake and debris clearance, was significantly reduced at the mRNA level in hiPSC-derived microglia exposed to OPN alone (*p* = 0.0362), LPS alone (*p* = 0.0388), and their combination (*p* = 0.0325), compared to untreated control cells [Welch’s ANOVA, W(3, 5.007) = 27.67, *p* = 0.0015; post-hoc power: 99.9%; Dunnett’s T3 *post hoc* test; Figure 5A].

At the same time, iNOS mRNA expression, a hallmark of antimicrobial activity and oxidative stress response, was increased only as a trend [*F* (3,12) = 3.051, *p* = 0.0699; Figure 5B]. Similarly, CD44, which has been shown to promote cytokine amplification and heightened inflammatory reactions in microglia, was not significantly altered by LPS or OPN at the mRNA level (Figure 5C).

All significant ANOVA results (viability, Sholl AUC, CD68 protein intensity, and CD206 mRNA) remained significant after Benjamini–Hochberg correction for multiple endpoint comparisons (FDR = 5%).

## Discussion

We confirmed and further characterized the microglial identity of hiPSC-derived microglia by demonstrating protein-level expression of key microglial markers and their close functional resemblance to *in vivo* microglia. Widely used murine microglia are known to differ in their gene expression profiles, activation states, and functionality. Species-specific differences include divergent transcriptional programs, limited genetic conservation, and distinct reactions to neurodegenerative conditions such as Alzheimer’s disease (Zerbino et al., 2018; Geirsdottir et al., 2019; Zhou et al., 2020). Therefore, translating scientific findings from murine cells to the human brain remains challenging due to interspecies disparities. Immortalized human microglia, as an alternative to primary or hiPSC-derived microglia, have been shown to lack key myeloid and microglia-specific markers such as IBA1 and PU.1 (Woolf et al., 2025). They exhibit less ramified morphology, likely reflecting a more activated basal state, and display reduced phagocytic activity and an attenuated cytokine response compared to hiPSC-derived microglia (Gao et al., 2024; Woolf et al., 2025). The transformation processes used for immortalization likely disrupt normal cellular regulation, thereby limiting their physiological functionality (Warden et al., 2023; Woolf et al., 2025).

In our cultures, hiPSC-derived microglia demonstrated a native CNS-specific immunophenotype at the protein level, expressing IBA1, TMEM119, PU.1, and SALL1, whereas Myb and MS4A7 associated with a peripheral myeloid phenotype were absent. These markers were selected to reflect the unique ontogeny of microglia from Myb-independent yolk sac progenitors, which characteristically express the identity- and function-defining factors SALL1 and PU.1. In contrast, Myb and MS4A7 are indicative of hematopoietic stem cell-derived peripheral macrophages and distinguish these cells from physiological embryonic microglia (Buttgereit et al., 2016; Buchrieser et al., 2017; Sonn et al., 2022; Bastos et al., 2025). To validate antibody specificity, THP-1 macrophages were stained under identical conditions and confirmed to be positive for Myb and MS4A7, while being negative for SALL1. This comparison supports the specificity of the negative staining results observed in hiPSC-derived microglia.

Upon exposure to the bacterial endotoxin LPS, hiPSC-derived microglia adopted rounded, process-retracted morphology by retracting their processes, demonstrating that our hiPSC-derived microglia respond with morphological changes consistent with microglia *in vivo* (Stence et al., 2001; Nimmerjahn et al., 2005). This morphology is currently understood to reflect heightened plasticity and adaptation to pathological or stimulatory cues and is associated with enhanced phagocytosis, migration, and cytokine release (Dyne et al., 2022; Green and Rowe, 2024; Vidal-Itriago et al., 2022).

The morphological modulation was accompanied by a significant increase in CD68, a marker of lysosomal and phagocytic activity in immune cells, indicative of a phagocytic or debris-clearing phenotype (Hopperton et al., 2018; Swanson et al., 2023). Conversely, CD68 mRNA expression remained unchanged, suggesting that protein-level regulation may occur independently of transcription, potentially through increased translation, trafficking, or protein stability (Hickman et al., 2013; Davis and Lloyd, 2024). Similar discrepancies between CD68 mRNA and protein expression have also been reported in non-myeloid cell types (Gottfried et al., 2008). Moreover, mRNA–protein dynamics have not been systematically characterized in hiPSC-derived microglial models, raising the possibility that these cells exhibit distinct intracellular regulatory kinetics compared to primary myeloid cells (Stansley et al., 2012). CD68 expression provides indicative evidence of enhanced lysosomal marker abundance, as CD68 is a well-established surrogate marker associated with the lysosomal compartment and phagocytic machinery. However, the unresolved mRNA–protein discrepancy requires clarification, for example, by functional assays or transcriptomic profiling. This would allow assessing biological function beyond protein expression. While preliminary, these findings offer a foundation for future studies by providing indicative evidence of enhanced lysosomal marker expression in hiPSC-derived microglia.

At the transcriptional level, hiPSC-derived microglia downregulated CD206, a marker associated with microglial states involving phagocytosis, tissue remodeling, and debris clearance, upon activation with various agents (A-Gonzalez et al., 2017).

These observations are consistent with the emerging view that microglial states exist along a continuum of context-dependent phenotypes rather than fitting into binary classifications such as M1/M2 (Ransohoff, 2016; Giovannoni and Quintana, 2020; Linnerbauer et al., 2020; Masuda et al., 2020). Recent studies emphasize that multimodal assays, including morphological, transcriptomic, metabolomic, and epigenetic analyses, are essential to fully capture these states (Paolicelli et al., 2022; Gao et al., 2025).

The present study employed morphological analysis, identity markers, and mRNA and protein expression, providing a descriptive foundation but not permitting definitive classification of microglial states. Nonetheless, these results can generate functional hypotheses regarding the immunological response of microglia to OPN stimulation.

We propose that OPN may restrain the transition to a fully reactive morphological state, in line with its proposed role in tissue repair, neuroregeneration, and resolution. These results underscore earlier findings that a supraphysiological inflammatory stimulus, such as LPS, induces a more pronounced loss of microglial ramification than a low-grade inflammatory or stress-related form of activation (Sugama et al., 2009). Furthermore, OPN prevented LPS-induced microglial cell death (compare Figure 4).

Importantly, our hiPSC-derived microglia not only responded to a classical endotoxin stimulation (LPS) but also to a physiological, endogenously produced modulator of immune activity in the CNS, namely OPN (Wang et al., 1998; Schroeter et al., 2006; Choi et al., 2007; Kang et al., 2008; Vay et al., 2021). Unlike LPS, which represents a pathogen-associated molecular pattern not typically encountered in the healthy brain, OPN is highly relevant to human neuroinflammation and is rapidly upregulated following CNS injuries such as ischemic stroke and multiple sclerosis relapses (Bird, 2007; Choi et al., 2007; Wang and Denhardt, 2008; Sun et al., 2024). By characterizing how human microglia respond to OPN, our study aims to provide insights into the functional reactive profile of microglia in response to endogenous, non-pathogen-derived injury signals. OPN did not exert any direct toxic effects on hiPSC-derived microglia at any tested concentration. However, hiPSC-derived microglia exhibited a reduction in process complexity in the presence of OPN, but not to the same extent as those exposed to LPS (compare Figure 1). Intriguingly, when hiPSC-derived microglia exposed to LPS were additionally treated with OPN, the toxic effects of LPS were attenuated (compare Figure 4) and the morphological effects were reduced (compare Figure 1), suggesting that OPN modulates cytoskeletal remodeling and limits LPS-induced structural simplification. This is consistent with previous findings in primary murine microglia, where OPN was shown to support microglial integrity and functional responsiveness under inflammatory or injury-related conditions (Rabenstein et al., 2016).

Overall, our data suggest that OPN is a physiological modulator of early microglial responsiveness, inducing a distinct, intermediate phenotype characterized by morphological preservation, increased CD68 intensity, and reduced homeostatic gene expression, without eliciting robust inflammatory signaling. Moreover, prior studies indicate indirect OPN-mediated regulation of CD44 through integrin signaling pathways, while the mechanistic linkage between OPN and CD44 in microglia remains incompletely understood. This may reflect a tailored response to sterile CNS injury, enabling microglia to prepare for debris clearance and tissue surveillance while minimizing collateral inflammation.

Notably, hiPSC-derived microglia responded to OPN—a brain-endogenous, context-relevant ligand—reinforcing the value of these cells for studying human neuroimmune interactions.

Future studies are warranted to see whether these responses are preserved or enhanced when hiPSC-derived microglia are placed in organotypic or 3D brain-like environments (Lancaster et al., 2013; Szebényi et al., 2021). Embedding microglia into brain organoids may allow for the emergence of additional context-dependent features, such as directed migration, cytokine signaling, or synaptic pruning, and could help reveal the full scope of OPN’s role in microglial function during injury and repair.

### Limitations of the study

Several limitations of this study should be acknowledged. First, while microglial identity was robustly demonstrated at the protein level using a discriminatory marker panel and validated with THP-1 macrophages as positive controls, this was a single-modality approach. For a broader multimodal perspective, flow cytometry or transcriptomic profiling would further refine the assessment of cellular purity and provide population-level molecular characterization. Second, CD68 intensity was used as a surrogate marker of lysosomal and phagocytic activity; however, direct functional assays such as fluorescent bead uptake or cytokine secretion measurements would be required to confirm functional consequences of the observed protein-level changes. The discrepancy between CD68 mRNA and protein levels remains unresolved and may reflect post-transcriptional regulatory mechanisms that warrant further investigation. Third, the mechanistic relationship between OPN and its putative receptors, CD44 and integrins, was not directly assessed in this study. Prior research has demonstrated OPN-mediated regulation of CD44 expression through integrin signaling pathways, as well as loss of CD44-dependent functions following OPN knockout in macrophages (Marroquin et al., 2004; Zhu et al., 2004). Receptor-level perturbation experiments would be necessary to establish causality in hiPSC-derived microglia. Finally, hiPSC-derived microglia, while recapitulating key features of brain-resident microglia, may exhibit subtle phenotypic differences compared to primary human microglia isolated from brain tissue (Paolicelli et al., 2022). The morphological and marker-based observations presented here provide descriptive evidence of microglial responses and should be interpreted as hypothesis-generating findings that warrant validation through multimodal analyses beyond staining techniques.

## Conclusion

We demonstrated that hiPSC-derived microglia expressed markers characteristic of native microglia, signifying their mesodermal origin and development along the myeloid lineage, while allowing a clear distinction from neuroepithelial-derived glial cells and peripheral macrophages. Our findings align with and extend previous characterization of microglia using a highly discriminatory marker panel, including a clear distinction from peripheral macrophages. hiPSC-derived microglia robustly responded to the endogenous immune modulator OPN, adopting an intermediate phenotype characterized by altered morphology, increased CD68 intensity, and downregulation of homeostatic markers. These responses differed from endotoxin-induced morphological changes and attenuated LPS-induced cell death. By highlighting OPN’s role as a physiological priming signal, our findings support its relevance in human neuroinflammation and emphasize hiPSC-derived microglia as a model for studying regulators of microglial activation.

## Data Availability

The raw data supporting the conclusions of this article will be made available by the authors, without undue reservation.

## Glossary

AUC: area under the curve
CD44: cluster of differentiation 44
CD68: cluster of differentiation 68
CD206: cluster of differentiation 206 (also known as MRC1)
CNS: central nervous system
EB: embryoid body
FACS: fluorescence-activated cell sorting
GM-CSF: granulocyte-macrophage colony-stimulating factor
hiPSC: human induced pluripotent stem cell
IBA1: ionized calcium-binding adapter molecule 1
IL-3: interleukin-3
IL-34: interleukin-34
iNOS: inducible nitric oxide synthase (gene name: NOS2)
LPS: lipopolysaccharide
M-CSF: macrophage colony-stimulating factor
MS4A7: membrane-spanning 4-domains subfamily A member 7
Myb: myeloblastosis oncogene
OPN: osteopontin (also known as secreted phosphoprotein 1, SPP1)
PFA: paraformaldehyde
PI: propidium Iodide
PU.1: transcription factor PU.1 (gene name: SPI1)
qPCR: quantitative polymerase chain reaction
ROCK: rho kinase
RPL13a: ribosomal protein L13a (housekeeping gene)
SALL1: spalt-like transcription factor 1
SSEA4: stage-specific embryonic antigen 4
TMEM119: transmembrane protein 119
TRA-1-81: tumor rejection antigen 1-81
