# Supplementary figures and images for: Microglia derived from human induced pluripotent stem cells are regulated by osteopontin, an endogenous extracellular matrix protein maintaining immune homeostasis
Source: Front Neurosci. 2026 Jun 24;20:1785992. doi: 10.3389/fnins.2026.1785992 (PMC13342188; doi:10.3389/fnins.2026.1785992)

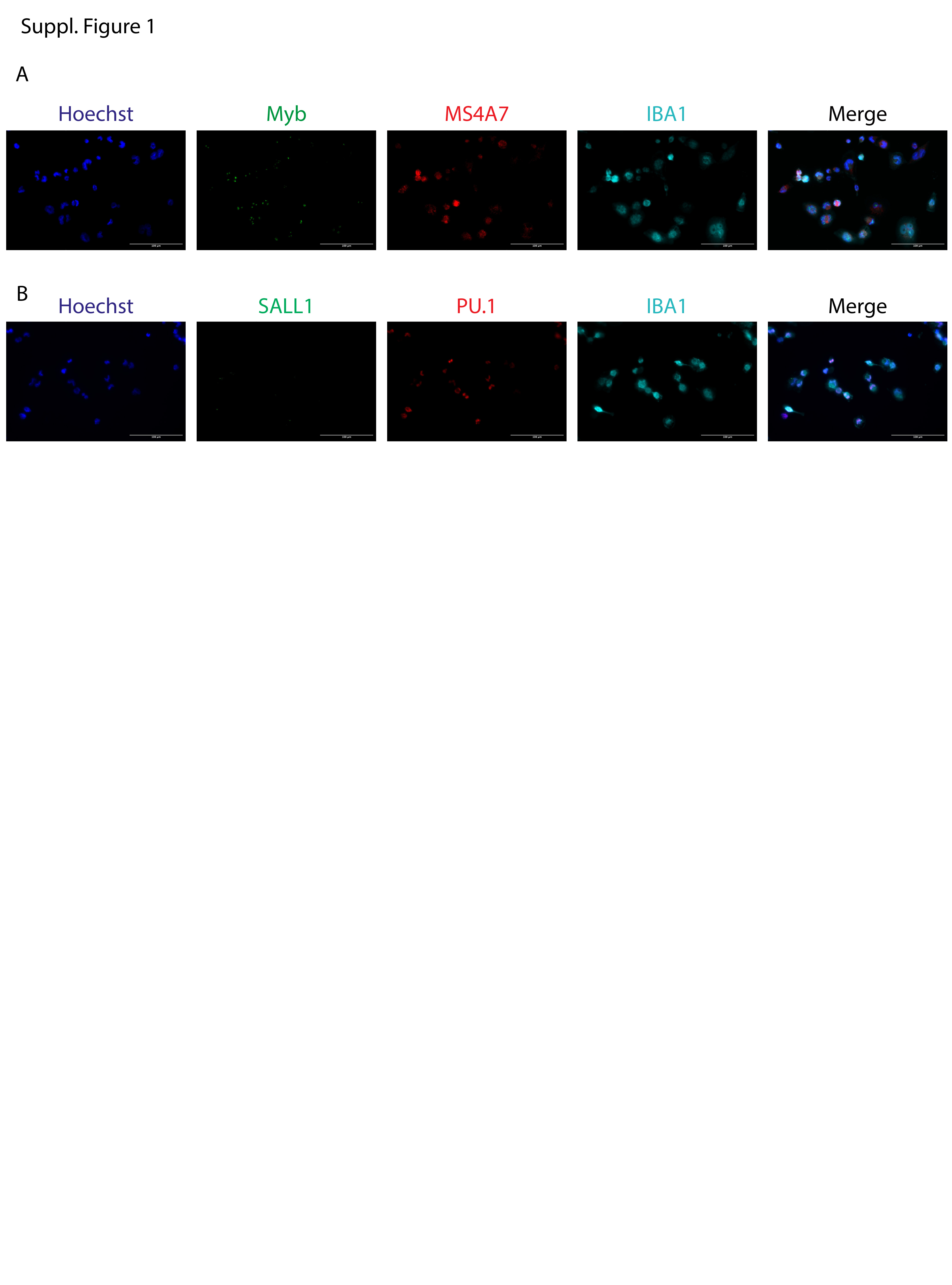

Supplement: Supplementary file 1 [file Image_1.jpg]
